# Human Airway Epithelial Cell Line Responses to Cigarette Smoke Extract and LPS: Insights Into Mucus and Cytokine Regulation

**DOI:** 10.1111/fcp.70117

**Published:** 2026-11-09

**Authors:** Aude Bodin, Tatiana Victoni, Yann Verres, Pascale Bellaud, Anne Casset, Alain Fautrel, Thomas Gicquel, Françoise Pons, Vincent Lagente

**Affiliations:** ^1^ NuMeCan Institute (Nutrition, Metabolism and Cancer) Univ Rennes, INSERM, INRAE, CHU Rennes Rennes France; ^2^ University of Lyon, VetAgro Sup Laboratory APCSe Marcy l'Étoile France; ^3^ H2P2 (Histopathology High precision) Platform, Univ Rennes Rennes France; ^4^ Université de Strasbourg, CNRS CAMB UMR 7199 Strasbourg France

**Keywords:** A549 and Calu‐3, COPD, CSE, mucus, NCI‐H292

## Abstract

**Background:**

Airway epithelial cells produce mucus to protect themselves against toxic agents and pathogens. However, excessive mucus accumulation, as seen in chronic obstructive pulmonary disease (COPD), contributes to airway obstruction, inflammation, and reduced efficacy of inhaled therapies.

**Objectives:**

The aim of the study was to develop an in vitro model for evaluating cytokine production and mucus secretion in human airway epithelial cell lines, with potential application for drug screening.

**Methods:**

Three human airway epithelial cell lines (A549, Calu‐3, and NCI‐H292) were stimulated for 24 h with cigarette smoke extract (CSE), alone or combined with lipopolysaccharide (LPS). In parallel, NCI‐H292 and Calu‐3 cells were co‐cultured under the same conditions. MUC5AC, CXCL8/IL‐8, CXCL1/GRO‐α, and CCL2/MCP‐1 expression was assessed by RT‐qPCR, and protein secretion by ELISA. MUC5AC production in NCI‐H292 was further evaluated by immunohistochemistry.

**Results:**

In Calu‐3 cells, stimulation had no significant effect on mucin or cytokine expression. In A549 cells, CSE + LPS induced a marked release of CXCL8/IL‐8, CXCL1/GRO‐α, and CCL2/MCP‐1, but no mucus production. In NCI‐H292 cells, LPS alone increased mucus production, and CSE + LPS had an additive effect on both mucins and cytokines (except CCL2/MCP‐1). In NCI‐H292/Calu‐3 co‐cultures, CSE + LPS enhanced MUC5AC gene expression, whereas LPS alone increased CXCL8/IL‐8 secretion.

**Conclusion:**

Among the tested cell lines, NCI‐H292 displayed the most consistent dual response in terms of mucus and cytokine production. This supports its use as a practical in vitro model for preliminary screening of therapies targeting airway inflammation and mucus hypersecretion.

**Abbreviations**
B[a]P: benzo [a] pyrene
BSA: bovine serum albumin
CCL: C‐C motif ligand
COPD: chronic obstructive pulmonary disease
CSE: cigarette smoke extract
CXCL: C‐X‐C motif ligand
DMSO: dimethyl sulfoxide
DNA: deoxyribonucleic acid
EDTA: ethylene diamine tetra acetic acid
EGFR: epidermal growth factor receptor
ELISA: enzyme linked immunosorbent assay
FBS: fetal Bovine Serum
GRO: growth regulated oncogene
Ig: immunoglobulin
IL: interleukin
LPS: lipopolysaccharide
MCP: monocyte chemoattractant protein
MUC: mucin
NF‐κB: nuclear factor‐kappa B
PBS: phosphate buffered saline
PMA: phorbol‐12‐myristate‐13‐acetate
RPMI: Roswell Park Memorial Institute medium
RT‐qPCR: real‐time quantitative polymerase chained reaction
TNF: tumor necrosis factor

## 1 Introduction

Chronic obstructive pulmonary disease (COPD) has become the 3rd leading cause of mortality and the 5th of morbidity worldwide. It is generally characterized by irreversible airflow obstruction, persistent airway inflammation and mucus secretion associated with immune cell infiltration in the lungs [1, 2].

Mucus secretion is essential for proper mucociliary function and airway homeostasis. It forms the first line of innate immune defense by trapping inhaled pathogens and toxic particles, which are subsequently expelled via ciliary beating (mucociliary clearance) [3]. However, this protective mechanism can become pathological when mucus accumulates and obstructs the airways. Airway mucus is primarily composed of mucin proteins, encoded by multiple MUC genes [3]. To date, 21 *MUC* genes in two families (secreted mucins and tethered mucins) have been identified [3, 4]. The main airway mucins are MUC5AC, produced by goblet cells, and MUC5B, secreted by submucosal glands [3, 4, 5]. Excessive mucus production in COPD is partly due to goblet cell hyperplasia and submucosal gland hypertrophy, leading to abnormally high levels of MUC5AC and MUC5B in the airways [3, 6, 7, 8].

Cigarette smoke is the major risk factor for COPD and has been shown to induce MUC5AC expression and protein production [9, 10, 11, 12]. Functionally, MUC5AC contributes to airway obstruction and hyperresponsiveness, whereas MUC5B plays a key role in maintaining effective mucociliary clearance [13]. Furthermore, bacterial infections during COPD exacerbations induce the secretion of mucus and also mediators, such the chemokines CXCL8/IL‐8, CXCL1/GRO‐α, and CCL2/MCP‐1 [6, 14], which recruit inflammatory cells to the site of inflammation [15, 16].

Given the global burden of COPD, there is a pressing need to identify new therapeutic targets. Gene therapy offers the potential for local and long‐term treatment, but its effectiveness is limited by poor vector uptake, partly due to the mucus barrier. Excessive mucus production further reduces the efficacy of inhaled therapies [17]. Therefore, developing a cost‐effective, time‐efficient, and reproducible in vitro model to test treatments targeting both mucus and inflammation is highly desirable.

Although numerous studies have examined the effects of stimuli such as cigarette smoke extract (CSE) or lipopolysaccharide (LPS) on mucus and cytokine production, few have directly compared multiple airway epithelial cell types under the same experimental conditions. The present study aimed to compare the responsiveness of three human airway epithelial cell lines—A549, NCI‐H292, and Calu‐3—differing in their capacity to produce mucus. Cells were exposed to low‐dose CSE, LPS from *Escherichia coli* , or both, and assayed for mucus and inflammatory mediator production in order to identify the most suitable in vitro model for evaluating both airway inflammation and the potential efficacy of nucleic acid vectors.

## 2 Materials and Methods

### 2.1 Reagents

Phosphate‐buffered saline (PBS), RPMI 1640 medium, penicillin–streptomycin, L‐glutamine, sodium pyruvate, and trypsin–EDTA were purchased from Life Technologies (Eugene, OR, USA). Fetal calf serum (FCS) was obtained from Hyclone (Logan, UT, USA), bovine serum albumin was from Eurobio (Les Ulis, France), and *E. coli* 055:B5 LPS was from Sigma‐Aldrich (St. Louis, MO, USA). A specific primary antibody against human MUC5AC (clone 45 M1) was purchased from Abcam (Cambridge, MA, USA), and a peroxidase‐coupled anti‐mouse IgG1 secondary antibody was purchased from Jackson ImmunoResearch Laboratories (West Grove, PA, USA).

### 2.2 Cell Culture

The A549 alveolar epithelial cell line, the NCI‐H292 bronchial epithelial cell line (derived from a mucoepidermoid carcinoma), and the Calu‐3 epithelial cell line (derived from a lung adenocarcinoma metastatic site; pleural effusion) were purchased from the American Type Culture Collection (Manassas, VA, USA). A549 and NCI‐H292 cells were cultured in RPMI 1640 medium, and Calu‐3 were cultured in α‐MEM medium. Media were supplemented with 10% FCS, 1% antibiotics, 2 mM L‐glutamine, 1 mM sodium pyruvate, and 10 mM HEPES. All cells were grown at 37°C and 5% CO_2_ in culture flasks containing complete culture medium. For experiments, they were transferred to culture plates (mucus and inflammatory mediator production) or Lab‐Tek chamber slides (Thermofisher Scientific, Rockford, IL, USA; immunohistochemistry experiments) as monoculture or co‐culture. For the monocultures, cells were seeded in 24‐well plates at a density of 2.25 × 10^4^ cells/cm^2^(A549) or 3.0 × 10^4^ cells/cm^2^(NCI‐H292), and 4.4 × 10^4^ cells/cm^2^ (Calu‐3). For the NCI‐H292 + Calu‐3 cell co‐culture, 5.5 × 10^4^ cells/cm^2^NCI‐H292 cells and 3.7 × 10^4^ cells/cm^2^Calu‐3 cells were seeded in 12‐well plates. For immunohistochemistry experiments, NCI‐H292 cells were seeded on Lab‐Tek chamber slides at a density of 2.0 × 10^4^ cells/cm^2^. All cultures were grown to 90%–100% confluence for 3 days. Cultures were starved in culture medium without serum for 24 h before exposure to CSE, LPS, or CSE + LPS.

### 2.3 CSE and Cell Treatments

CSE was prepared by bubbling the smoke from two 2R1 cigarettes (Center for Tobacco Reference Products, Lexington, KY, USA) through 20 mL of RPMI medium. The resulting suspension (100% CSE) was filtered through a 0.2 μm filter, and diluted with complete medium as previously described [14]. A549, NCI‐H292, and Calu‐3 cells were stimulated with CSE (2%, 4%, 8%, or 10%) with or without LPS (0.1 or 1 μg/mL) or with LPS alone for 24 h (mRNA and protein measurements) or 48 h (protein measurements). At the end of the incubation period, culture supernatants were harvested and stored at −80°C until further analysis. All experiments were performed in triplicate in serum‐free medium and were repeated at least three times. Incubation of each cell line with CSE alone, LPS alone, or both for 24 and 48 h did not affect cell viability as assessed by the ATP cell assay (Supplementary Figure 1).

### 2.4 Cytokine Assay

The concentrations of CXCL8/IL‐8, CXCL1/GRO‐α, and CCL2/MCP‐1 in the culture supernatant were measured using Duoset ELISA kits (R&D Systems, Abingdon, UK), according to the manufacturer's instructions. For the MUC5AC assay, supernatants were incubated in 96‐well ELISA plates (R&D Systems, Abingdon, UK), according to the manufacturer's instructions.

### 2.5 MUC5AC Assay

For the MUC5AC assay, microtiter plates (R&D Systems, Abingdon, UK) were coated with 100 μL of cell culture supernatant or cell lysate, overnight at 40°C and blocked with 200 μL of PBS containing 0.05% Tween and 2% BSA for 1 h at room temperature. Next, the plates were successively incubated with a specific mouse anti‐human MUC5AC antibody (1 to 2 μg/mL), a horseradish peroxidase‐coupled anti‐mouse IgG1 secondary antibody (dilution: 1/5000) and the peroxidase substrate, 3,3′,5,5′‐tétraméthylbenzidine. The absorbance intensity at 450 nm was measured spectrophotometrically with a POLARstar Omega (BMG labtech, Ortenberg, Germany) microplate reader. Sample results were expressed as fold changes relative to the absorbance measured for culture medium alone or PBS alone.

### 2.6 Quantitative Reverse Transcription PCR Analysis

After cell lysis, total RNA was isolated using a NucleoSpin RNAII kit (Macherey‐Nagel, Dueren, Germany), according to the manufacturer's protocol. Total RNA (1 μg) was reverse‐transcribed into first‐strand cDNA using a High‐Capacity cDNA Achieve Kit (Applied Biosystems, Foster City, CA, USA), according to the manufacturer's instructions. RNA quantity and purity were assessed with a Nanodrop ND‐1000 spectrophotometer (Nyxor Biotech, Paris, France). Quantitative real‐time PCRs (RT‐qPCRs) were then performed using the fluorescent dye SYBR Green method, with SYBR Green PCR Master Mix in 384‐well plates on an ABI PRISM 7900HT system (Applied Biosystems, Foster City, CA, USA). Primer pairs for each transcript were chosen with NCBI software (www.ncbi.nlm.nih.gov): *MUC5AC* forward 5′‐TGG AGC TGG ACA CCA AAT AC‐3′, reverse 5′‐CTT GGT GTT GTG GGA GAG G‐3′; *MUC5B* forward 5′‐GAA CTG CCA GAG CTG TAA CT‐3′, reverse 5′‐CAT CGG TGG TGT AGA TGA‐3; *CXCL8* (*IL‐8*) forward 5′‐AAG AAA CCA CCG GAA GGA AC‐3′, reverse 5′‐AAA TTT GGG GTG GAA AGG TT‐3′, *CXCL1* (*GRO‐α*) forward 5′‐AAC CGA AGT CAT AGC CAC AC‐3′, reverse 5′‐CCT CCC TTC TGG TCA GTT G‐3′; *CCL2* (*MCP‐1*) forward 5′‐TGT CCC AAA GAA GCT GTG ATC‐3′, reverse 5′‐ATT CTT GGG TTG TGG AG TGA G‐3′; *GAPDH* forward 5′‐ATG ACA TCA AGA AGG TGG TG‐3′, reverse 5′‐CAT ACC AGG AAA TGA GCT TG‐3′. Human *GAPDH* was used as the reference gene. Amplification curves were read with the QuantStudio Real‐Time PCR Software (Applied Biosystems, Foster City, CA, USA), using the comparative cycle threshold method. The relative steady‐state mRNA level was normalized against *GAPDH* mRNA.

### 2.7 Immunocytochemistry for MUC5AC

Immunocytochemical staining was performed on the Discovery Automated IHC stainer using the Ventana OMNIMap detection kit (Ventana Medical Systems, Tucson, Ariz). After exposure to CSE, LPS, or CSE + LPS, Lab‐Tek chamber slides were immersed in Carnoy's solution for 2 h at 4°C and permeabilized with BSA‐saponin 0.5% for 30 min at room temperature. Then, endogenous peroxidase was blocked with Inhibitor‐D 3% H_2_O_2_ (Ventana) for 12 min at 37°C. After rinsing with reaction buffer (Roche), slides were incubated at 37°C for 60 min with 1/50 dilution of mouse monoclonal Mucin 5 AC. After rinsing, signal enhancement was performed using the anti‐Mouse HRP immunoglobulin IgG(H + L) secondary antibody (Ventana) for 16 min and Ventana OmniDABMap Kit for 44 min. Slides were then counterstained for 16 min with hematoxylin, 4 min with bluing reagent, and rinsed. After removal from the instrument, slides were manually dehydrated and coverslipped. MUC5AC staining was quantified with NIS‐Elements software (Nikon Instruments Inc., Melville, NY, USA). For each condition, three independent slides were analyzed. Three randomly selected areas were examined on each slide, and the images presented are representative of the entire slide. For each experimental condition, a representative image was acquired at 20 × magnification. Lower‐magnification images acquired at 5 × and without magnification are provided as supplementary data (Supplementary Figure 3 and Supplementary Figure 4).

### 2.8 Statistical Analysis

Statistical analyses were performed using Graph Pad Prism 9. Data were expressed as the mean ± standard error of the mean (SEM). Intergroup differences as a function of the treatment were probed in a one‐way analysis of variance (ANOVA). The Kruskal–Wallis test was used to compare the different stimuli. In all analyses, the threshold for statistical significance was set to *p* < 0.05.

## 3 Results

### 3.1 Effects of LPS or/and CSE on MUC5AC Gene Expression and Protein Secretion

We first assessed MUC5AC gene expression and protein secretion in three airway epithelial cell lines and in the NCI‐H292/Calu‐3 co‐culture after exposure to LPS, CSE, or their combination.

In A549 cells, only low‐dose LPS for 24 h moderately upregulated MUC5AC mRNA (Figure 1A), without affecting protein secretion (Figure 1E). Calu‐3 cells showed no change in mRNA or protein levels at any LPS dose (Figure 1B–F). In contrast, NCI‐H292 cells exhibited increased MUC5AC mRNA and protein secretion at all LPS concentrations (Figure 1C,G). In the co‐culture, LPS increased MUC5AC mRNA but did not alter protein secretion (Figure 1D–H).

**FIGURE 1:**
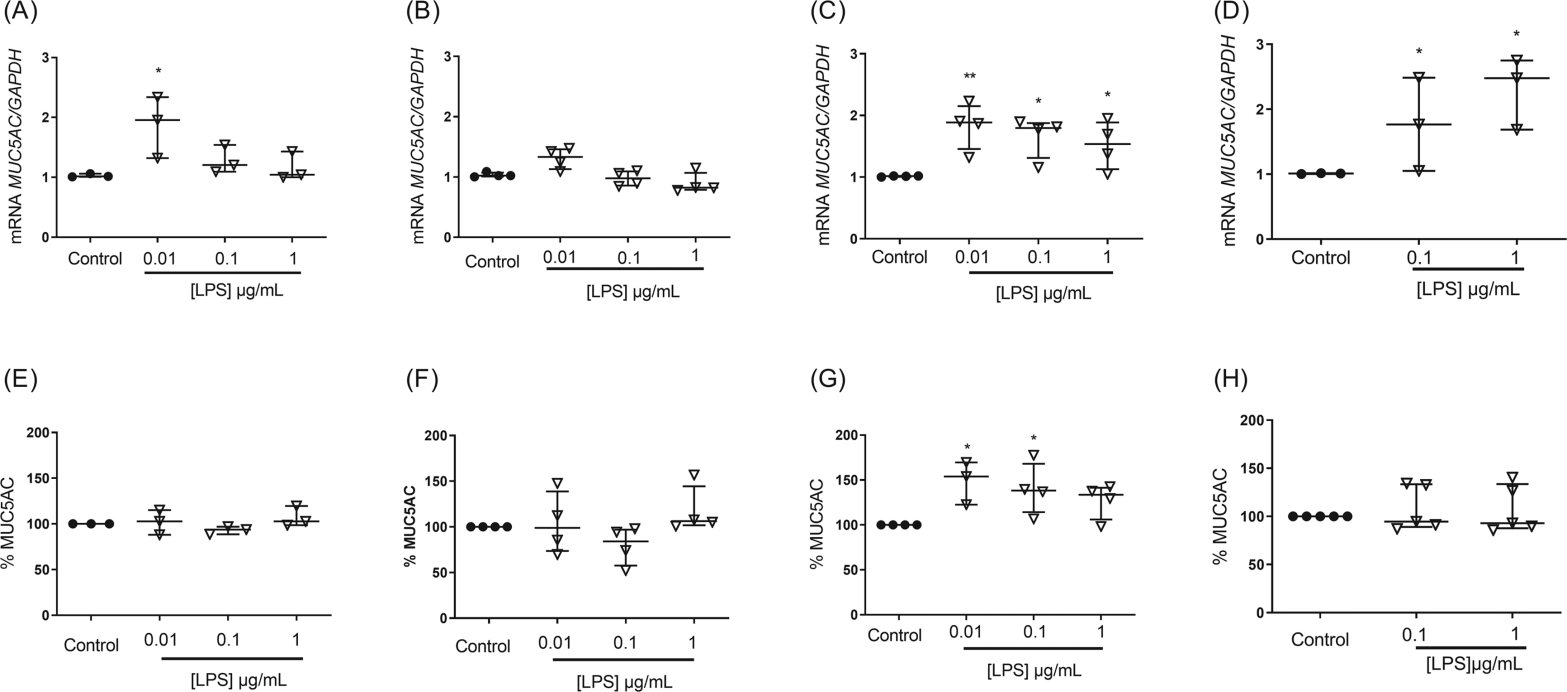
Comparison of relative MUC5AC mRNA expression levels (measured using a RT‐qPCR) and protein secretion levels (measured using an ELISA) after exposure of A549 cells (A and E), Calu‐3 cells (B and F), NCI‐H292 cells (C and G), and an NCI‐H292‐Calu‐3 co‐culture (D and H) to increasing doses of LPS for 24 h. The data represent the mean ± SEM of three to four experiments. For graphs A to D, *MUC5AC* mRNA expression was normalized against *GAPDH*. For graphs E to H, the values correspond to the colorimetric intensity (i.e., the amount of MUC5AC protein) relative to those measured after exposure to medium alone (the control, set to 100%). The threshold for statistical significance was set to *p* < 0.05 (*p < 0.05; **p < 0.01 versus the control).

Next, we studied *MUC5AC* gene expression after exposure to CSE. A549 and Calu‐3 cells were unresponsive in terms of MUC5AC mRNA and protein (Figure 2A,B,E,F). In NCI‐H292 cells, CSE induced a dose‐dependent increase in MUC5AC mRNA, without affecting protein secretion (Figure 2C,G). In the co‐culture, only the highest CSE doses elevated MUC5AC mRNA, with no change in protein levels (Figure 2D–H).

**FIGURE 2:**
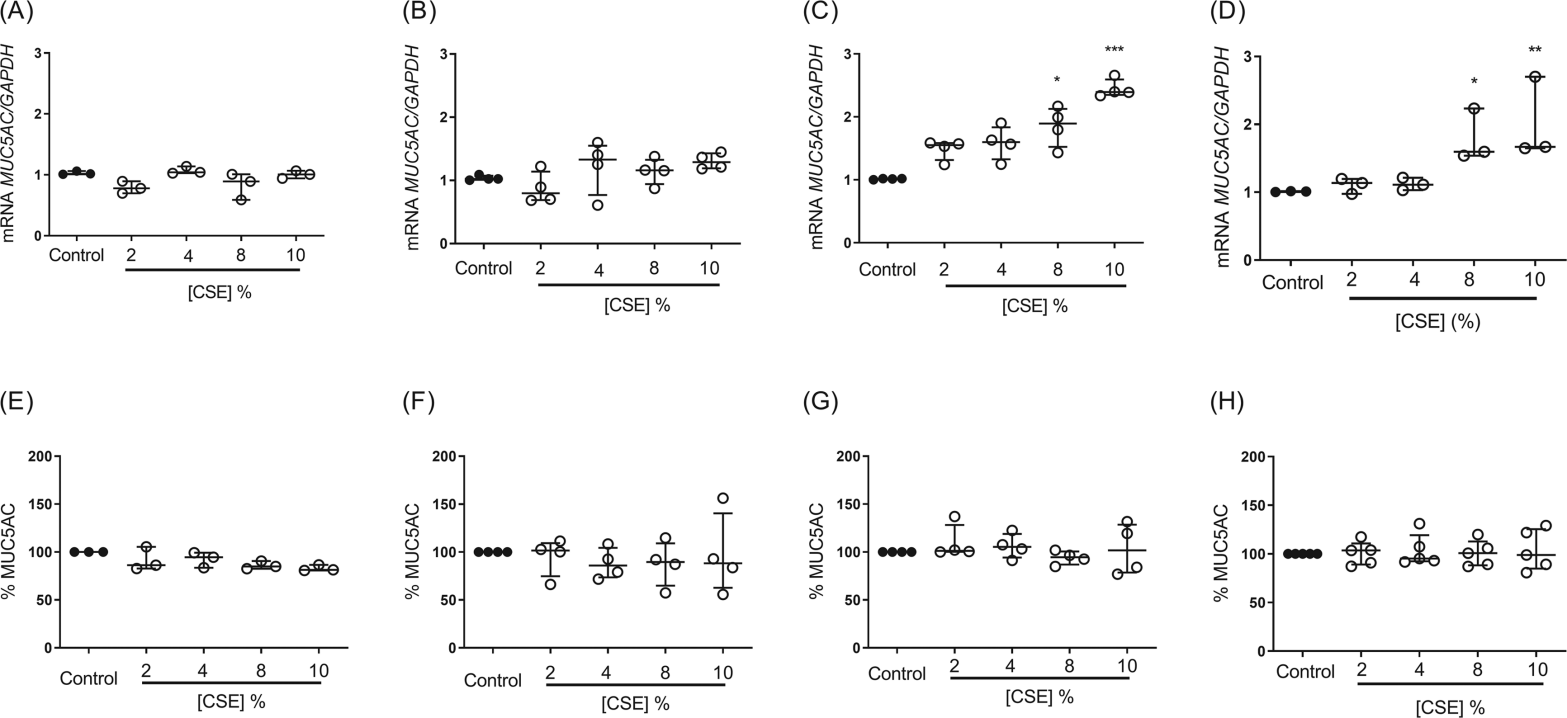
Comparison of relative MUC5AC mRNA expression levels (measured using a RT‐qPCR) and protein secretion levels (measured using an ELISA) after exposure of A549 cells (A and E), Calu‐3 cells (B and F), NCI‐H292 cells (C and G), and an NCI‐H292‐Calu‐3 co‐culture (D and H) to increasing doses of CSE for 24 h. The data represent the mean ± SEM of three to four experiments. For graphs A to D, *MUC5AC* mRNA expression was normalized against *GAPDH*. For graphs E to H, the values correspond to the colorimetric intensity (i.e., the amount of MUC5AC protein) relative to those measured after exposure to medium alone (the control, set to 100%). The threshold for statistical significance was set to *p* < 0.05 (*p < 0.05; **p < 0.01).

Lastly, we exposed the cells to a combination of 4% CSE and 0.1 μg/mL LPS for 24 h. For A549 and Calu‐3 cells, the combination of these stimuli did not result in any additional effect on MUC5AC mRNA and protein levels, whether in terms of secretion or intracellular production (Figure 3A,B and Supplementary Figure 2). NCI‐H292 cells showed an additive increase in MUC5AC mRNA relative to single treatments, with a modest increase in protein secretion or production intracellularly (Supplementary Figure 2), though not significantly higher than CSE or LPS alone (Figure 3A,B). In the co‐culture, the combination did not significantly alter MUC5AC mRNA or protein levels compared to individual stimuli (Figure 3A,B).

**FIGURE 3:**
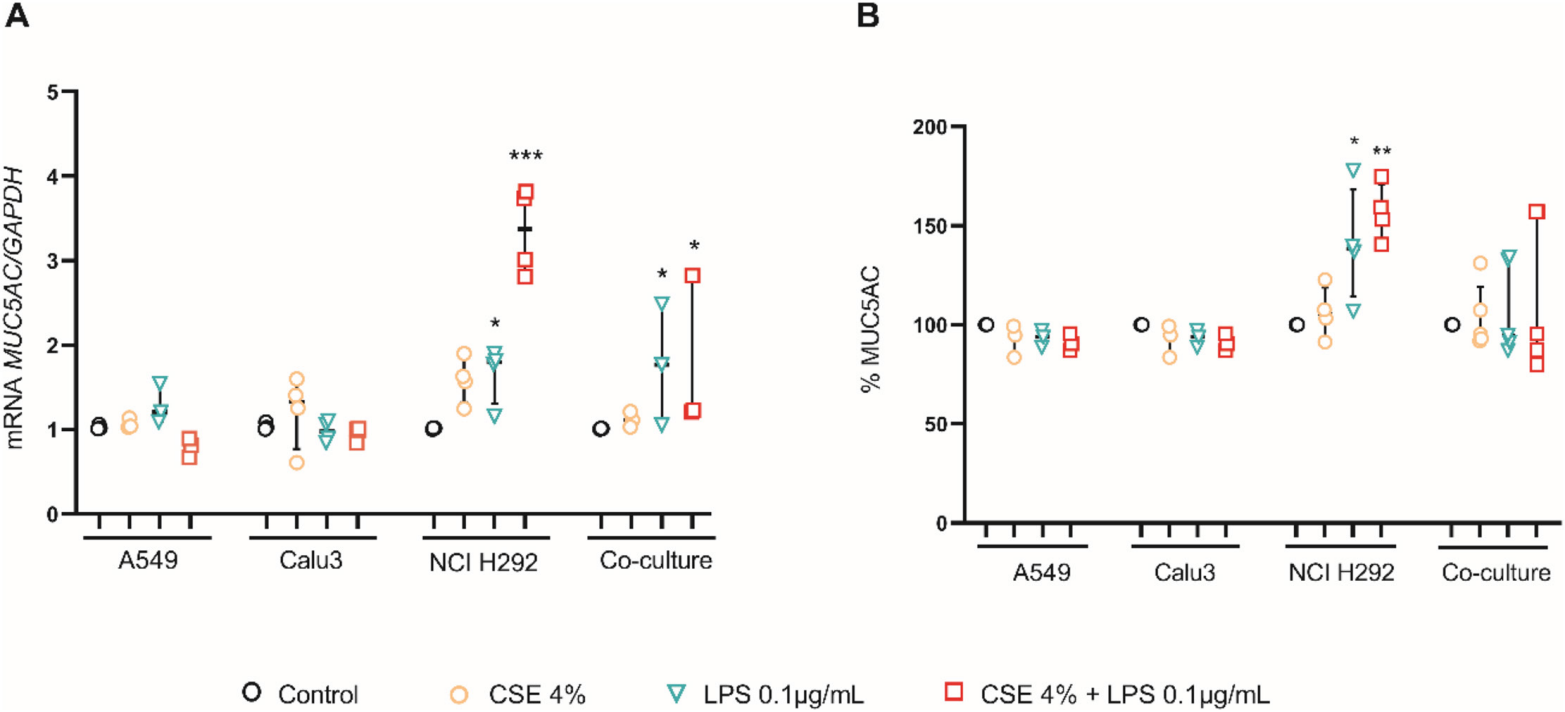
Comparison of MUC5AC expression levels (measured using a RT‐qPCR) and protein secretion levels (measured using an ELISA) after exposure to a combination of 4% CSE and 0.1 μg/mL LPS for 24 h. The graphs represent *MUC5AC* gene expression (A) and protein secretion (B). Data represent the mean ± SEM of three to four experiments. For graph A, *MUC5AC* mRNA expression was normalized against *GAPDH*. For graph B, the values correspond to the colorimetric intensity (i.e., the amount of MUC5AC protein) relative to those measured after exposure to medium alone (the control, set to 100%). The threshold for statistical significance was set to *p* < 0.05 (*p < 0.05; **p < 0.01; ***p < 0.001 versus control).

### 3.2 Effects of LPS or/and CSE on Gene Expression and Secretion of CXCL8/IL‐8, CXCL1/GROα, and CCL2/MCP1

We next examined the inflammatory response by measuring mRNA and protein levels of CXCL8/IL‐8, CXCL1/GRO‐α, and CCL2/MCP‐1. In A549 cells and the co‐culture, CSE + LPS significantly increased mRNA levels of CXCL8/IL‐8 (Figure 4A), whereas Calu‐3 cells remained unresponsive. NCI‐H292 cells responded to LPS alone, with further elevation when combined with CSE. Protein secretion reflected mRNA patterns, with A549 showing low secretion under LPS alone, Calu‐3 and co‐culture exhibiting high baseline secretion, and NCI‐H292 increasing secretion only in response to LPS (Figure 4B).

**FIGURE 4:**
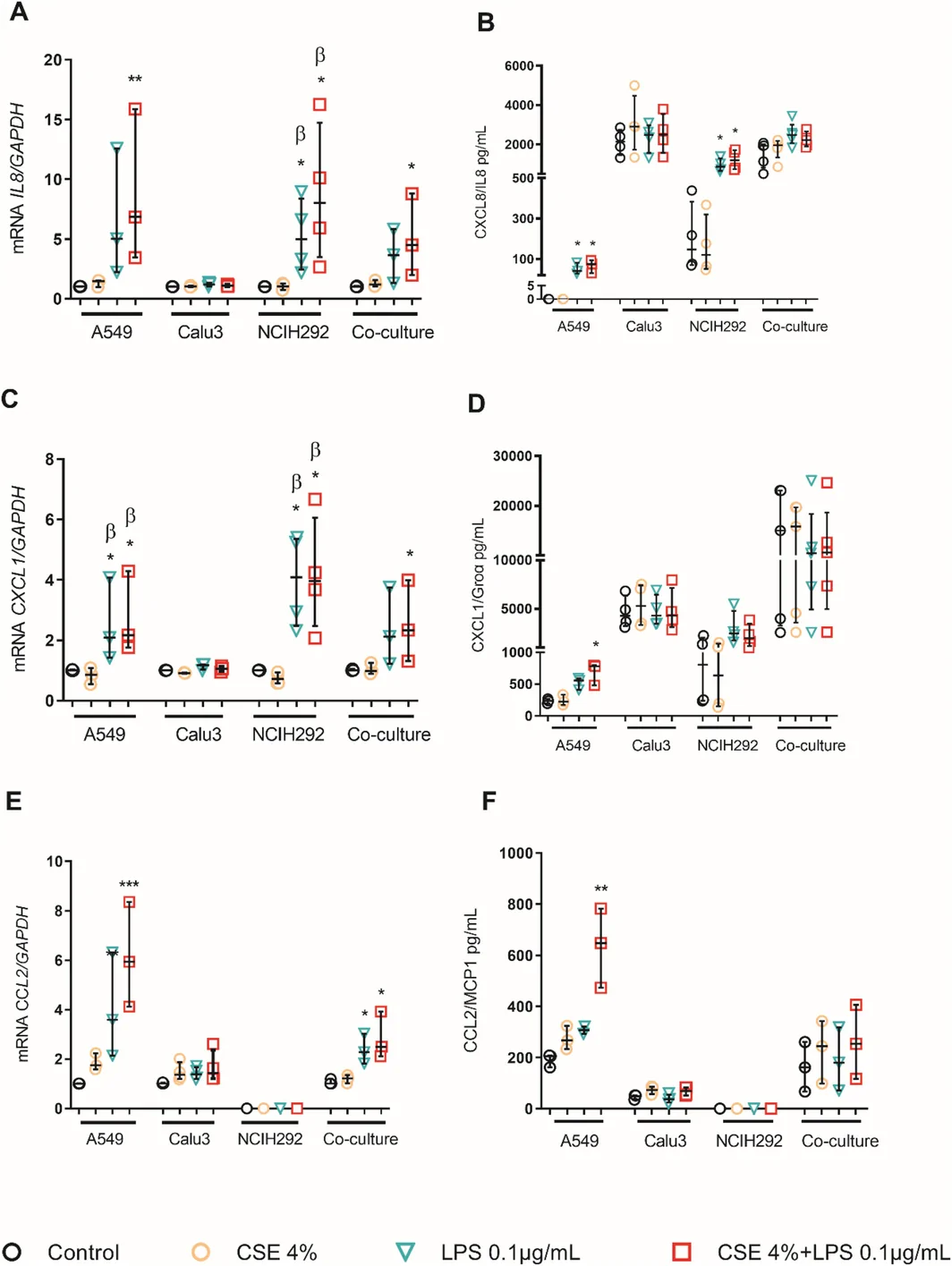
Comparison of chemokine mRNA levels and secreted protein levels after exposure to CSE, LPS, or both for 24 h. Chemokine protein secretion was quantified in an ELISA. The graphs represent *CXCL8/IL‐8* gene expression (A) and protein secretion (B), *CXCL1/Gro‐α* gene expression (C) and protein secretion (D), and *CCL2/MCP‐1* gene expression (E) and protein secretion (F) by the three cell lines and the co‐culture. Data represent the mean ± SEM of three to four experiments. The mRNA expression for each chemokine was normalized against *GAPDH*. The threshold for statistical significance was set to *p* < 0.05 (*p < 0.05; **p < 0.01; ***p < 0.001 versus control; β *p* < 0.05 versus CSE).

In regard to CXCL1/GRO‐α, LPS and CSE + LPS increased CXCL1 mRNA in A549, NCI‐H292, and the co‐culture, while Calu‐3 remained unchanged (Figure 4C). Protein elevation was observed only in A549 exposed to CSE + LPS; high baseline secretion in Calu‐3 and co‐culture masked further increases (Figure 4D).

Finally, for CCL2/MCP‐1, NCI‐H292 did not express CCL2/MCP‐1. Calu‐3 showed no mRNA changes. In A549 and the co‐culture, LPS increased CCL2/MCP‐1 mRNA, without additive effect from CSE. Significant protein secretion was observed in A549 cells only following combined CSE + LPS exposure, whereas Calu‐3, NCI‐H292, and the co‐culture showed no detectable response. (Figure 4E,F).

### 3.3 Immunohistocytochemistry for MUC5AC in the NCI H292 Cell Line

Given that NCI‐H292 was the only line responding consistently in both mucus and cytokine production, we performed immunohistochemical staining for MUC5AC after 24 h exposure to 4% CSE, 0.1 μg/mL LPS, or their combination. Under control conditions, MUC5AC levels were low. Neither CSE nor LPS alone significantly altered staining, whereas the combination induced increased MUC5AC production, confirming RT‐qPCR and ELISA results (Figure 5A–E). We also measured MUC5B expression in NCI‐H292 cells. LPS alone upregulated MUC5B mRNA, with a further increase when combined with CSE (Figure 5F).

**FIGURE 5:**
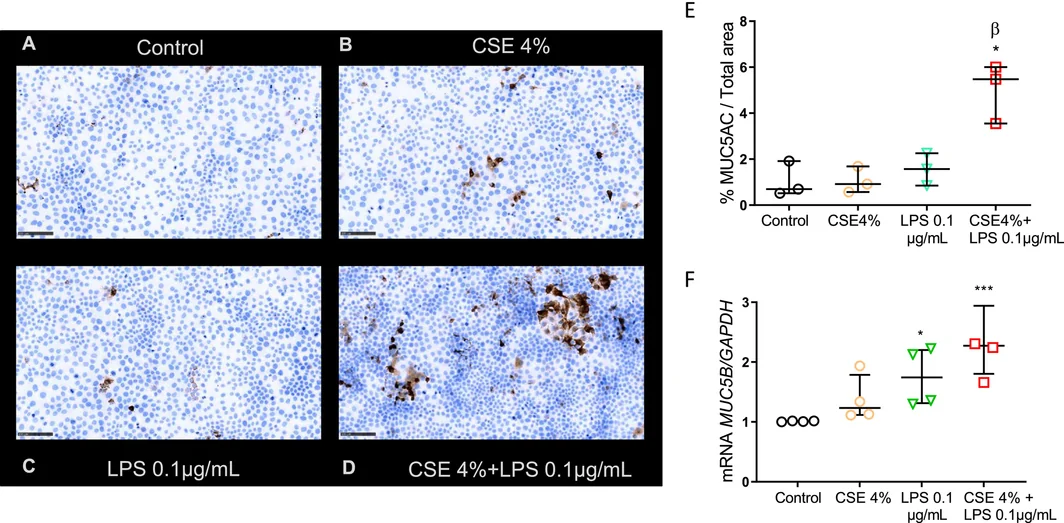
Induction of MUC5AC production by NCI‐H292 cells. Cells were cultured without treatment (A) or exposed to 4% CSE (B), 0.1 μg/mL LPS (C), or both (D) for 24 h. After fixation, the cells were labeled with an anti‐MUC5AC antibody, and the latter was revealed with DAB. For each experimental condition, a representative image was acquired at 20 × magnification. (E) The MUC5AC staining area, from three independent experiments. The data represent the mean ± SEM MUC5AC area as a percentage of the total area. (F) Induction of *MUC5B* mRNA levels after exposure of NCI‐H292 cells to 4% CSE and/or 0.1 μg/mL LPS for 24 h. Data represent the mean ± SEM from three to four experiments. *MUC5B* mRNA expression was normalized against *GAPDH*. The threshold for statistical significance was set to *p* < 0.05 (*p < 0.05; **p < 0.01; ***p < 0.001 versus control; β *p* < 0.05 versus CSE).

## 4 Discussion

Excessive mucus production is a hallmark of respiratory diseases such as COPD [17]. This mucus barrier not only contributes to airway obstruction but also reduces the efficacy of inhaled therapies, including gene therapy. Therefore, developing a simple, cost‐effective in vitro model to evaluate treatments targeting both mucus and inflammation is highly desirable. Although several studies have investigated the effects of stimuli such as CSE or LPS on mucus and cytokine production, few have directly compared the responses of multiple airway epithelial cell types under the same conditions [18, 19, 20, 21, 22, 23].

The main objective of this work was to analyze the response of bronchial epithelial cells in terms of inflammatory mediator and mucus production. COPD affects both the proximal airways (inflammation, mucus hypersecretion) and the distal alveoli (destruction, emphysema). We therefore aimed to identify one cell line particularly sensitive to inflammatory stimuli, another more prone to mucus production, and to combine the two in order to analyze the associated responses. In this context, we chose to work with:

A549, a type II pneumocyte line, suitable for studying alveolar processes such as surfactant production, inflammation, and oxidative stress; Calu‐3, derived from proximal bronchi, which form a functional epithelial barrier and secrete mucus; NCI‐H292, of tracheal/bronchial origin, widely used as a reference model for mucin hyperproduction, particularly MUC5AC. Other immortalized cell lines generated with SV40 or HPV (such as BEAS‐2B and 16HBE14o‐) are closer to normal cells than tumor‐derived lines. However, they are of bronchial origin and thus do not produce surfactant. BEAS‐2B cells are mainly used for toxicological studies of inhaled substances, while 16HBE14o‐ cells are particularly valuable for investigating epithelial barrier function and CFTR‐dependent ion transport. Consequently, these cells were not retained for our study, as they are less relevant in the context of COPD, which involves both alveolar and bronchial compartments.

We then compared three airway epithelial cell lines (A549, NCI‐H292, and Calu‐3) and a co‐culture system to low doses of CSE and/or LPS. Among these, NCI‐H292 cells emerged as the most suitable model, exhibiting a robust response in terms of mucus production, particularly following combined exposure to 4% CSE and 0.1 μg/mL LPS. This combination also induced elevated mRNA expression of both MUC5AC and MUC5B, while LPS alone increased secretion of inflammatory mediators (CXCL8/IL‐8 and CXCL1/GRO‐α).

Our findings are consistent with previous reports showing that NCI‐H292 cells upregulate mucin gene expression and protein production in response to various stimuli, including CSE, benzo[a]pyrene (B[a]P), tumor necrosis factor α (TNFα) or phorbol‐12‐myristate‐13‐acetate (PMA) [18, 19, 20, 23, 24]. In agreement with Lin et al., we observed that LPS alone increased MUC5AC expression and secretion, with CSE exerting an additive effect. Similarly, the induction of CXCL8/IL‐8 and CXCL1/GRO‐α by LPS aligns with prior studies, while the lack of CCL2/MCP‐1 secretion in NCI‐H292 cells confirms previous observations that this chemokine is not produced under particulate or CSE stimulation [25]. Finally, MUC5B expression was elevated upon LPS exposure and further enhanced by CSE, consistent with the literature suggesting distinct regulatory pathways for MUC5B versus MUC5AC secretion [18, 21, 22, 25]. The researchers suggested that the secretion of MUC5B protein does not involve the same EGFR‐dependent MAPK Erk1/2 signaling pathway as the secretion of MUC5AC [26].

A549 cells, representing type II alveolar epithelial cells, showed a limited mucin response but retained the ability to secrete inflammatory chemokines, particularly when exposed to the CSE + LPS combination. These results contradict the report by Di et al., in which exposure to CSE led to elevated levels of *MUC5AC* mRNA [9]. The duration of CSE exposure in Di et al.'s study was 2 h—suggesting that a short‐term induction of *MUC5AC* mRNA induction reverts to baseline during a 24 h exposure. Furthermore, it has been reported that exposure to LPS from *Pseudomonas aeruginosa* is associated with elevated *MUC5AC* gene expression [27], whereas we found that low doses (but not high doses) of LPS from *E. coli* induced mucin mRNA expression. Nonetheless, other literature data indicate that the A549 cell line is less sensitive to CSE than primary human bronchial epithelial cells [28] and NCI‐H292 cells [25]. Although secreted protein levels were lower than those for the other two cell lines, the A549 cell line appeared to produce an inflammatory response (CXCL8/IL‐8, CXCL1/GRO‐α, and CCL2/MCP‐1 secretion) following stimulation with a combination of LPS and CSE [29].

Our results showed that exposure to CSE and/or LPS did not appear to influence the activation of Calu‐3 tracheobronchial epithelial cell line; mRNA levels of the three chemokines and the mucin in stimulated cells did not differ significantly from the control value. Indeed, we observed that the baseline levels of cytokine and MUC5AC secretion were already quite high, our results suggested that these cells are activated in the absence of treatment. The Calu‐3 cell line has been described as a model of the bronchial epithelium, with mucus secretion and tight junction formation—especially when cultured at an air‐liquid interface [30]. It has also been reported that exposure to acrolein (an irritant component of cigarette smoke) was associated with elevated MUC5AC production by the Calu‐3 cell line [31]. Another study found that exposure of the Calu‐3 cell line to LPS from *P. aeruginosa* is associated with elevated *MUC5AC* gene transcription and elevated CXCL8/IL‐8 secretion [32]. In the present study, LPS from *E. coli* did not appear to activate Calu‐3 cells. Thus, we hypothesize that submerged cultures of Calu‐3 cells do not respond optimally to CSE and/or LPS, suggesting that air‐liquid interface (ALI) culture may be necessary to fully activate these cells.

The NCI‐H292/Calu‐3 co‐culture system was designed to mimic the epithelial barrier by combining mucus‐producing and cytokine‐secreting cells. While gene expression was elevated in response to CSE + LPS, protein secretion did not increase significantly, indicating that the co‐culture did not substantially alter the functional response compared to NCI‐H292 alone.

This study has limitations. First, mucin quantification via ELISA may be less precise than mass spectrometry, but this method was chosen to maintain a cost‐effective and practical screening model. Second, immortalized cell lines do not fully replicate primary human bronchial epithelial cell (HBEC) physiology. Future studies using HBECs cultured at ALI could better assess inhaled therapies, including gene delivery vectors, and provide a more physiologically relevant model, though at higher cost and complexity.

In conclusion, NCI‐H292 cells stimulated with CSE and LPS represent a practical and responsive in vitro model for evaluating mucus production and inflammatory responses. This model provides a rapid and reproducible platform to test the efficacy of novel therapies, including gene therapy vectors, targeting airway inflammation and mucus hypersecretion. Future work using primary bronchial epithelial cells under ALI conditions could further enhance the physiological relevance and translational potential of this approach.

## Author Contributions

Conception and study design: Aude Bodin, Tatiana Victoni, Vincent Lagente, and Françoise Pons. Data acquisition: Aude Bodin, Anne Casset, and Yann Verres. Data analysis: Aude Bodin, Pascale Bellaud, Alain Fautrel, and Tatiana Victoni. Manuscript drafting: Aude Bodin, Tatiana Victoni, Vincent Lagente, and Françoise Pons. Manuscript revising: Aude Bodin, Tatiana Victoni, Vincent Lagente, Françoise Pons, Anne Casset, Thomas Gicquel, and Alain Fautrel. All authors have read and approved the final version of this manuscript to be published.

## Funding

This study was supported by ANR Project Luther and INSERM (France).

## Ethics Statement

The authors have nothing to report.

## Conflicts of Interest

The authors declare no conflicts of interest.

## Supporting information


**Supplementary Figure 1** **Cell viability**. Cell viability assessed by ATP assay in A549, Calu‐3, NCI‐H292, and NCI‐H292–Calu‐3 co‐cultures after 24 h exposure to increasing concentrations of CSE and/or LPS. Data are presented as mean ± SEM from three to four independent experiments. Statistical significance was defined as *p* < 0.05 versus control.
**Supplementary Figure 2** Comparison of MUC5AC protein production levels (measured using an ELISA) after exposure to 4% CSE combined or not with 0.1 μg/mL LPS for 24 h. The graphs represent MUC5AC protein production. Data represent the mean ± SEM of three to four experiments. The values correspond to the colorimetric intensity (i.e., the amount of MUC5AC protein) relative to those measured after exposure to PBS alone (the control, set to 100%). The threshold for statistical significance was set to *p* < 0.05 (*p < 0.05; **p < 0.01; ***p < 0.001 versus control).
**Supplementary Figure 3** Induction of MUC5AC production by NCI‐H292 cells. Cells were cultured without treatment (A) or exposed to 4% CSE (B), 0.1 μg/mL LPS (C), or both (D) for 24 h. After fixation, the cells were labeled with an anti‐MUC5AC antibody, and the latter was revealed with DAB. For each experimental condition, a representative image was acquired at 5 × magnification.
**Supplementary Figure 4** Induction of MUC5AC production by NCI‐H292 cells. Cells were cultured without treatment (A) or exposed to 4% CSE (B), 0.1 μg/mL LPS (C), or both (D) for 24 h. After fixation, the cells were labeled with an anti‐MUC5AC antibody, and the latter was revealed with DAB. For each experimental condition, a representative image was acquired but without magnification.

## Data Availability

The data that support the findings of this study are available from the corresponding author upon reasonable request.
