# Supplementary material for: Human Airway Epithelial Cell Line Responses to Cigarette Smoke Extract and LPS: Insights Into Mucus and Cytokine Regulation
Source: Fundam Clin Pharmacol. 2026 Nov 9;40(6):e70117. doi: 10.1111/fcp.70117 (PMC13748795; doi:10.1111/fcp.70117)
Supplement: Supplementary file 1 [file FCP-40-0-s001.docx]

**Supplementary data**

**Supplementary Figure 1**

**Supplementary Figure 1: Cell viability.** Cell viability assessed by ATP assay in A549, Calu-3, NCI-H292, and NCI-H292–Calu-3 co-cultures after 24 h exposure to increasing concentrations of CSE and/or LPS. Data are presented as mean ± SEM from three to four independent experiments. Statistical significance was defined as p < 0.05 versus control.

**Supplementary Figure 2**

**Supplementary Figure 2:** Comparison of MUC5AC protein production levels (measured using an ELISA) after exposure to 4% CSE combined or not with 0.1 µg/mL LPS for 24 hours. The graphs represent MUC5AC protein production. Data represent the mean ± SEM of three to four experiments. The values correspond to the colorimetric intensity (i.e. the amount of MUC5AC protein) relative to those measured after exposure to PBS alone (the control, set to 100%). The threshold for statistical significance was set to *p*<0.05 (* p<0.05; ** p<0.01; *** p<0.001 versus control).

**Supplementary Figure 3**

**Supplementary Figure 3:** Induction of MUC5AC production by NCI-H292 cells. Cells were cultured without treatment (A) or exposed to 4% CSE (B), 0.1 µg/mL LPS (C), or both (D) for 24 hours. After fixation, the cells were labeled with an anti-MUC5AC antibody, and the latter was revealed with DAB. For each experimental condition, a representative image was acquired at 5× magnification.

**Supplementary Figure 4**

**(A)**

(B)

(C)

(D)

**Supplementary Figure 4:** Induction of MUC5AC production by NCI-H292 cells. Cells were cultured without treatment (A) or exposed to 4% CSE (B), 0.1 µg/mL LPS (C), or both (D) for 24 hours. After fixation, the cells were labeled with an anti-MUC5AC antibody, and the latter was revealed with DAB. For each experimental condition, a representative image was acquired but without magnification.
